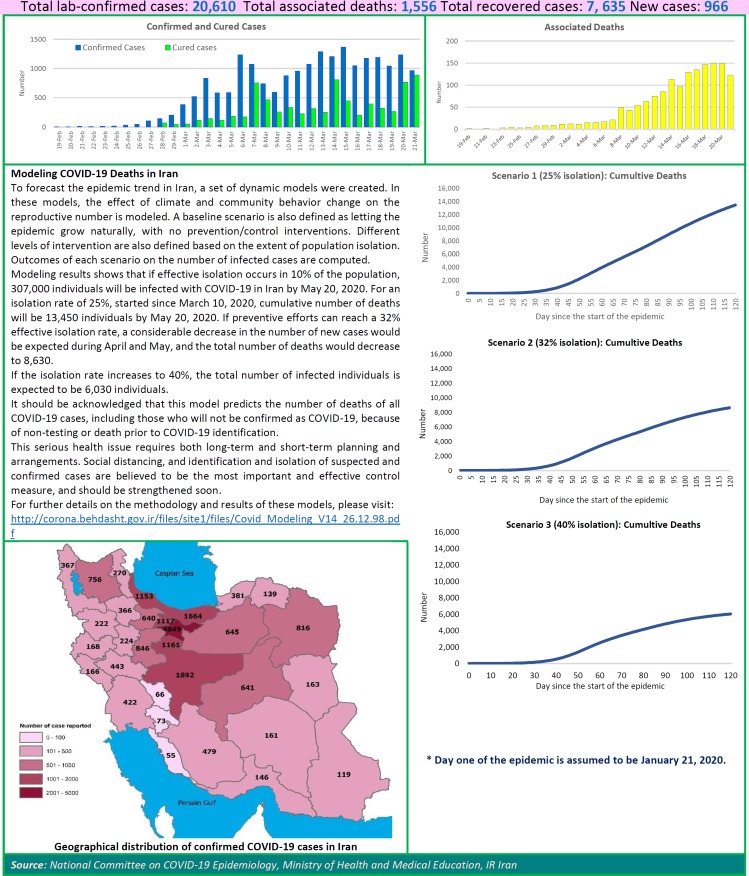# Daily Situation Report on Coronavirus disease (COVID-19) in Iran; March 22, 2020

**Published:** 2020-03-22

**Authors:** National Committee on COVID-19 Epidemiology, Ministry of Health and Medical Education, IR Iran

**Keywords:** COVID-19, severe acute respiratory syndrome coronavirus 2, Hospital Mortality, epidemiology, Pandemics, Health Information Exchange

## Abstract

To forecast the COVID-19 epidemic trend in Iran, a set of dynamic models were created. In these models, the effect of climate and community behavior change on the reproductive number is modeled. A baseline scenario is also defined as letting the epidemic grow naturally, with no prevention/control interventions. Different levels of intervention are also defined based on the extent of population isolation. Outcomes of each scenario on the number of infected cases are computed.

Modeling results shows that if effective isolation occurs in 10% of the population, 307,000 individuals will be infected with COVID-19 in Iran by May 20, 2020. For an isolation rate of 25%, started since March 10, 2020, cumulative number of deaths will be 13,450 individuals by May 20, 2020. If preventive efforts can reach a 32% effective isolation rate, a considerable decrease in the number of new cases would be expected during April and May, and the total number of deaths would decrease to 8,630. If the isolation rate increases to 40%, the total number of infected individuals is expected to be 6,030 individuals.

It should be acknowledged that this model predicts the number of deaths of all COVID-19 cases, including those who will not be confirmed as COVID-19, because of non-testing or death prior to COVID-19 identification.

This serious health issue requires both long-term and short-term planning and arrangements. Social distancing, and identification and isolation of suspected and confirmed cases are believed to be the most important and effective control measure, and should be strengthened soon. For further details on the methodology and results of these models, please visit:

http://corona.behdasht.gov.ir/files/site1/files/Covid_Modeling_V14_26.12.98.pdf

**Figure:**